# The Etiological and Predictive Association Between ADHD and Cognitive Performance From Childhood to Young Adulthood

**DOI:** 10.1177/10870547231159908

**Published:** 2023-04-11

**Authors:** Isabella Vainieri, Giorgia Michelini, Celeste H. M. Cheung, Olakunle A. Oginni, Philip Asherson, Frühling Rijsdijk, Jonna Kuntsi

**Affiliations:** 1 King’s College London, UK; 2 University College London, UK; 3 Queen Mary University of London, UK; 4 University of California Los Angeles, USA; 5 Education Endowment Foundation, London, UK; 6 Obafemi Awolowo University, Ile-Ife, Nigeria; Frühling Rijsdijk is now affiliated to Anton de Kom University of Suriname, Suriname, South America

**Keywords:** ADHD, cognitive impairments, etiological factors

## Abstract

**Objective:**

Evidence about the etiology of the predictive associations between a diagnosis of ADHD and cognitive performance over time is scarce. Here, we examine these predictive and etiological patterns using a cross-lagged model design in a sample of 404 participants (74% males) from ADHD and control sibling pairs aged 6 to 17 years at baseline and 12 to 24 years at follow-up.

**Methods:**

Data included IQ, short-term and working memory measures, and response speed and variability from a four-choice reaction-time task.

**Results:**

ADHD and IQ predicted each other over time. ADHD at baseline predicted lower working memory performance at follow-up. Stable etiological influences emerged in the association between ADHD and cognitive variables across time.

**Conclusion:**

Whether early interventions can reduce negative interference with learning at school requires further study.

## Introduction

Attention deficit hyperactivity disorder (ADHD) is a highly heritable disorder, characterised by developmentally inappropriate levels of inattention and hyperactivity-impulsivity (Faraone et al., 2021). ADHD affects around 5.9% of children and adolescents worldwide and often persists into adulthood, with significant impact on individuals’ everyday lives (Faraone et al., 2021). In addition to the symptoms of inattention and hyperactivity-impulsivity, ADHD is associated with cognitive impairments, including decreased vigilance and increased attention fluctuations (often measured with reaction time variability [RTV]), impairments in executive functions (e.g., inhibitory control and working memory), and on average, lower IQs (Franke et al., 2018). Cross-sectional studies indicate overall similar patterns of cognitive impairments associated with ADHD in childhood, adolescence, and adulthood (Franke et al., 2018).

Longitudinal studies using repeated measurements of cognitive impairments from childhood to adolescence or young adulthood have broadly shown that individuals with persistent ADHD display persisting impairments in RTV, IQ, and measures of inhibition and working memory (Cheung et al., 2016; Lin & Gau, 2019; Tallberg et al., 2021; Thissen et al., 2014). Findings from longitudinal studies that investigated whether early cognitive difficulties in childhood predict future ADHD outcomes have reported less consistent findings. Available population-based and clinical studies have found that childhood impairments in IQ and executive functions, such as inhibition and planning, predicted ADHD symptoms and diagnosis in children, adolescents, and young adults (Agnew-Blais et al., 2016; Berlin et al., 2003; Brocki et al., 2007; Campbell & von Stauffenberg, 2009; Cheung et al., 2015; Gao et al., 2015; Manfro et al., 2019; Miller et al., 2013; Shephard et al., 2022). Yet, mixed results have been reported for RTV and working memory, as these measures predicted later ADHD symptoms in adolescents and young adulthood in some studies (Kalff el., 2002; Karalunas et al., 2017; Sjöwall et al., 2015; van Lieshout et al., 2016) but not in others that assessed ADHD in early childhood and late adolescence (Berlin et al., 2003; Brocki et al., 2007; Campbell & von Stauffenberg, 2009; Cheung et al., 2015).

Possible explanations for these mixed findings are differences in study design and measures, including whether measurement of ADHD symptoms relied on parent or self-reports and whether functional impairment was part of the measurement. Also, these studies focused exclusively on investigating the prediction of later ADHD by examining early cognitive functioning, but there are different ways in which ADHD and cognitive functioning can influence each other over time. For instance, early ADHD may predict later cognitive impairments over time. In this case, we can hypothesise that the behavioral symptoms of ADHD in childhood may contribute to the worsening of later cognitive impairments. Evidence from longitudinal studies shows that high levels of ADHD symptoms in early childhood predict poor reading and mathematics skills, and lower executive functioning skills in childhood, adolescence, and young adulthood (O’Neill et al., 2017, 2016; Sasser et al., 2015; Schmiedeler & Schmiedeler, 2014). Alternatively, ADHD and cognitive impairments may have reciprocal effects and predict each other over time, indicating that not only would the early behavioral symptoms of ADHD contribute to the subsequent worsening of cognitive performance but also that early cognitive impairments would contribute to the worsening of ADHD symptoms over time. Finally, ADHD and cognitive impairments may be associated at both time 1 and time 2, but neither predicts each other, indicating that ADHD and cognitive impairments co-occur without influencing each other over time. Better understanding the direction of the association between ADHD and cognitive functioning over time could help to identify strategies to prevent negative long-term outcomes.

A cross-lagged model allows the simultaneous examination of longitudinal influences of one variable on another, and vice versa, while also controlling for concurrent associations between variables over time. Despite several studies having investigated the relationship between ADHD and cognitive performance over time, to our knowledge, only a few studies to date have applied the cross-lagged design to test this association (Arnett et al., 2012; Fan & Wang, 2022; Rajendran et al., 2013; Rommel et al., 2015). These studies showed reciprocal associations of ADHD symptoms with overall cognitive functioning in pre-schoolers followed up for 3 years (Rajendran et al., 2013), with speed processing measured with rapid naming speed between ages 4 and 5 years and ages 10 to 11 years (Arnett et al., 2012), with verbal and performance IQ between ages 12 and 16 years (Rommel et al., 2015), and with response inhibition (Fan & Wang, 2022). Overall, these studies showed that ADHD symptoms and cognitive functioning negatively impact each other across time, likely interfering in educational settings. Yet, since these studies were conducted on ADHD traits in population-based samples, it remains unclear whether these reciprocal associations also impact children and adolescents with clinical diagnoses of ADHD.

Twin and sibling studies enable us to move beyond observable phenotypic associations, to explore their etiology. Such quantitative genetic studies indicate substantial genetic and familial risk influences underlying the cross-sectional association between cognitive functioning and ADHD symptoms or diagnosis. Population-based twin studies in children show substantial overlap of genetic influences between ADHD symptoms and response speed (mean reaction time [MRT]), RTV, inhibition, working-memory performance (digit span backward [DSB]) and IQ (Kuntsi, Rogers, et al., 2006; Wood et al., 2011). Similarly, evidence from clinical sibling samples shows shared familial influences between ADHD and RTV, IQ, working memory, and short-term memory (measured with DSB and digit span forward [DSF]; Michelini et al., 2018; Wood et al., 2011). While the etiological associations between ADHD and cognitive impairments have been investigated cross-sectionally, evidence on the stability of the etiological influences that account for these associations over time is scarce. Only one study investigated the etiological association between ADHD symptoms and cognitive abilities, specifically focusing on IQ, in a population-based twin sample at 12, 14, and 16 years using a genetically informative cross-lagged design in a twin sample (Rommel et al., 2015). This study provided evidence that stable genetic influences underlie the association between ADHD symptoms and IQ from early to late adolescence (Rommel et al., 2015). To our knowledge, no study to date has investigated the association between ADHD and cognitive functioning, using a cross-lagged design, in clinically diagnosed individuals with ADHD while exploring the etiological factors involved in the association between ADHD diagnosis and cognitive processes over time. Such investigation can help elucidate the predictive and etiological patterns involved in the association between ADHD and cognitive performance across development, as well as point to potential opportunities for intervention.

The first aim of this study is to investigate longitudinally the direction of the association between ADHD diagnosis and cognitive performance that previously showed strong cross-sectional associations with ADHD using the same sample used in this study (IQ, DSF, DSB, and reaction time measures of RTV and MRT (Cheung et al., 2016; James et al., 2017; Michelini et al., 2016)). Using data from childhood and subsequent follow-up assessments in adolescence and young adulthood of 404 individuals from ADHD and control sibling pairs, we test the predictive association between ADHD and cognitive measures across the two time points. The second aim is to explore whether latent familial and non-familial influences that underlie ADHD diagnosis and cognitive measures, as well as the association between ADHD diagnosis and each cognitive impairment, are stable over time.

## Methods

### Participants

Participants aged between 6 and 17 years were recruited from specialist clinics in the UK from among those who had a clinical diagnosis of DSM-IV combined subtype ADHD during childhood. Closest-age siblings were also then recruited and assessed for ADHD. A control group, which was initially recruited from primary (ages 6–11 years) and secondary (ages 12–18 years) schools in the UK (Kuntsi et al., 2010) was also assessed at baseline and follow-up. Exclusion criteria applied at the initial childhood assessment included IQ < 70, autism, epilepsy, general learning difficulties, brain disorders, and any genetic or medical disorder associated with externalising behaviors that might mimic ADHD. The investigation was carried out in accordance with the latest version of the Declaration of Helsinki.

The sample retained at follow-up (on average 6 years after initial assessment) consisted of 404 participants. After exclusions of participants with missing data, the sample included 391 participants: 99 participants with persistent ADHD and 100 unaffected siblings (69 full sibling pairs, 61 singletons), 23 remitters (5 full sibling pairs, 13 singletons), and 169 control siblings (76 full sibling pairs, 17 singletons). Further information on the sample is given in Supplementary material. Participants whose ADHD diagnosis changed from baseline to follow-up were retained in the analyses to account for the stability and change in ADHD diagnosis over time.

There were no significant differences between the groups in age, but they differed significantly in sex, with more males in the ADHD group compared to unaffected siblings and controls and no females among remitters (Supplemental Table S1). A 48-hour ADHD medication-free period was required prior to assessments. All participants and parents provided informed consent. Study procedures were approved by the London-Surrey Borders Research Ethics Committee (09/H0806/58).

## Measures

### ADHD Diagnosis

At baseline the Parental Account of Childhood Symptoms (PACS; Taylor et al., 1986) interview was conducted with the parents to assess the 18 DSM-IV symptoms for ADHD index cases plus siblings who were thought, on the basis of parents’ descriptions of behavior or Conner’s scores of 65 or greater, to potentially have ADHD. Situational pervasiveness was defined as some symptoms occurring in two or more different situations from the PACS, as well as the presence of one or more symptoms scoring 2 or more from the DSM-IV ADHD subscale of the teacher-rated Conner’s subscale. Impairment criteria were based on the severity of symptoms identified in the PACS.

At follow-up, ADHD diagnostic status was assessed with the Diagnostic Interview for ADHD in Adults (DIVA; Ramos-Quiroga et al., 2019), a semi-structured interview designed to evaluate the DSM-IV criteria for childhood and adult ADHD. Evidence of impairments commonly associated with ADHD was assessed with the Barkley’s Functional Impairment Scale (BFIS; Barkley & Murphy, 2006), by trained researchers, along with the DIVA during face-to-face interviews with parents. Parent-report DIVA and impairments were used to determine ADHD status based on DSM-IV criteria.

### IQ, Digit Span Forward, and Digit Span Backward

IQ was measured using the vocabulary and block design subtests of the Wechsler Intelligence Scale for Children third edition (WISC-III; Wechsler, 1991) at baseline and using the vocabulary and block design subtests of the Wechsler Abbreviated Scale of Intelligence (WASI; Wechsler, 1999) ad follow-up. The digit span subtest from the WISC-III at baseline and WASI at follow-up were administered to obtain digit span forward (verbal short-term memory) and digit span backward (verbal working memory).

### The Fast Task

The task was a computerised four-choice reaction time (RT) task which measures performances under a slow-unrewarded and a fast-incentive condition (Kuntsi, Rogers, et al., 2006). The slow-unrewarded (baseline) condition consists of 72 trials, which followed a standard warned four-choice RT task. Four empty circles (warning signals, arranged horizontally) first appeared for 8 seconds, after which one of them (the target) was colored in. Participants were asked to press the response key that corresponded to the position of the target. Following a response, the stimuli disappeared from the screen and a fixed inter-trial interval of 2.5 seconds followed. Speed and accuracy were emphasised equally. A comparison condition that used a fast event rate (fore-period of 1 second) and incentives followed immediately after the baseline condition and consisted of 80 trials, with a fixed inter-trial interval of 2.5 seconds following the response. Participants were told to respond as quickly as possible to win smiley faces and real prizes (£5). The smiley faces appeared below the circles in the middle of the screen when participants responded faster than their own MRT during the baseline condition consecutively for three trials and were updated continuously. The variables obtained from the task were MRT and RTV (the standard deviation (*SD*) of reaction times from the baseline and fast-incentive condition).

### Statistical Analyses

A cross-lagged model enriched by analyses to fit sibling data was accomplished by structural equation model (SEM) using the OpenMx package in R (Boker et al., 2011). The model deals with missing data by calculating the log likelihood of the data for each observation using raw maximum likelihood estimation with 95% confidence intervals.

### Model-Fitting of Sibling Data

As siblings share on average 50% of their segregating genes and 100% of the common environment, we can decompose the variance/covariance of traits into contributions of familial influences (the combined effects of shared genetic and shared environmental effects) and non-familial influences (individual-specific effects and measurement error; Cheung et al., 2012; Kuntsi et al., 2010). Sibling-pair data allow us to derive: phenotypic correlations in each sibling, for example the correlation between IQ and ADHD, constrained across sibling order (first or second born); cross-sibling/within-trait correlations, for example the correlation between sibling 1 and sibling 2 for IQ; and cross-sibling/cross-trait correlations, constrained such that, for example the correlations between IQ in Sibling 1 and ADHD in Sibling 2 equals the correlation of IQ in Sibling 2 and ADHD in Sibling 1. The cross-sibling/within-trait and the cross-sibling/cross-trait correlations allow us to estimate, respectively, the familial variance of a trait and the familial overlap between traits.

A liability threshold model framework, which assumes that the liability of a disorder is underpinned by a normally distributed continuum of risk (Rijsdijk et al., 2005), was used as the binary ADHD affection status variable which was measured as present or absent. Given the selected nature of this sample (selection based on ADHD diagnosis), ADHD status was included in the model with its parameters fixed to population-known values based on previous evidence and consistent with our previous work (Cheung et al., 2012; James et al., 2016; Kuntsi et al., 2010; Michelini et al., 2018). Specifically, prevalence was fixed to 5% (Willcutt, 2012; *z* score set at 1.64); the cross-sibling/within-trait correlation (correlation between siblings in each pair) was fixed to 0.40 (Chang et al., 2013; Larsson et al., 2014); the familiality to 0.40 (representing 80% genetic variance in case of null shared environmental effects; Larsson et al., 2013); the correlation between ADHD at time 1 and at time 2 to 0.70 (Cheung et al., 2015; Rommel et al., 2015). For further explanation of this approach, see Rijsdijk et al. (2005). Since we are using cross-sectional family data to assess the relationships between data over time, possible sources of errors can inflate familial and non-familial estimates. To address this issue, measurement error (ME) was estimated for each predicting variable (Heath et al., 1993). ME for ADHD was fixed to 0.10 based on the high reliability (0.90) of parent-reported ADHD diagnosis (Zhang et al., 2005). Age and sex were controlled for in all analyses by regressing out their effects from continuous variables (before transforming to normality), as standard practice for family model-fitting studies (McGue & Bouchard, 1984) and in line with our previous work on the same sample (Cheung et al., 2012; James et al., 2016; Kuntsi et al., 2010; Michelini et al., 2018). To account for positive skewness, we applied appropriate transformations to all measures prior to analyses. Descriptive statistics for all study variables at both time points are available in Supplemental Table S2.

### Cross-Lagged Paths

The cross-lagged model allows the examination of the direction of the association between variables across time using partial regression coefficients (phenotypic cross-lagged and stability paths), while taking into account the pre-existing relationship between variables at baseline. Estimates are defined as small (<0.30), medium (0.30–0.50), and large (>0.50; Cohen, 1988). Separate analyses were conducted for each pair of variables (ADHD diagnosis with IQ, DSB, DSF, MRT in each task condition, and RTV in each task condition).

**Figure 1.**
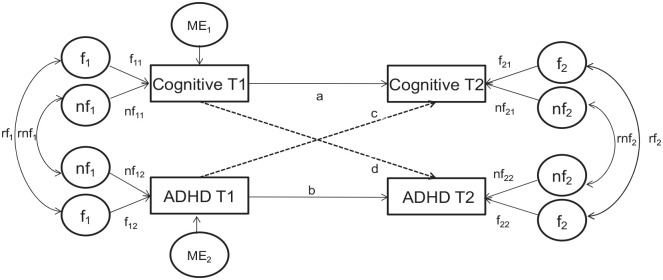
Cross-lagged model. *Note*. Familial and non-familial correlations are represented by r*f* and r*nf*, respectively. Phenotypic causal path coefficients are represented by: *a* and *b* for stability paths and *c* and *d* for cross lagged paths. If coefficient *c* is significant, ADHD at baseline predicts the cognitive measure at follow-up. If coefficient *d* is significant, the cognitive measure at baseline predicts ADHD at follow-up. If both *c* and *d* are significant, there is a reciprocal association between variables over time. *f* = familial effects; *nf* = non-familial effects; ME = measurement error; *T*1 = time 1; *T*2 = time 2.

The phenotypic cross-lagged paths (Figure 1) connect different measures across time points (paths *c* and *d*). The regression coefficients of the cross-lagged paths are used to examine the direction of the association between ADHD and each cognitive variable (IQ, DSF, DSB, and RTV and MRT in both task conditions) across time. The stability paths connect the same measure across time points (*a* and *b*) and represent the contribution of a variable at time 1 to the same variable at time 2 (e.g., the higher the coefficient, the higher the stability of a variable over time).

At each time point, the contribution of familial and non-familial influences on DSB, DSF, MRT in both task conditions, and RTV in both task conditions are calculated, giving an estimate of the time-specific familial and non-familial influences (Figure 1, outer sides: *f, nf*). Familial and non-familial influences on ADHD diagnosis were fixed at time 1 and 2. At time 2, familial and non-familial influences on each variable are estimated as residuals, indicating the familial and non-familial contributions independent of the familial and non-familial influences transmitted from time 1 (i.e., time-specific for time 2).

Time-specific familial and non-familial correlations between ADHD, and DSB, DSF, MRT, and RTV in both task conditions are estimated at each time point (Figure 1, outer sides: r*f*, r*nf*). Familial and non-familial correlations indicate the extent to which the familial and non-familial influences impacting on ADHD are the same as those impacting on one of the cognitive variables. At time 2, familial and non-familial influences on the correlations between variables are estimated as residuals, indicating the association between the variables independent of their relationship at time 1 (time-specific for time 2).

To examine the stability of familial and non-familial influences on the association between ADHD and cognitive variables over time, the covariance between ADHD and cognitive functioning was divided into covariance specific to time 2 (due to correlated residual factors; r*f*_2_ and r*nf*2) and covariance transmitted from time 1 (due to correlated familial and non-familial factors at time 1 and the cross-lagged and stability paths; r*f*_1_, r*f*_1_ and *a, b*, c, *d*, respectively). The transmitted covariance between ADHD and cognitive variable from time 1 (i.e., stable covariance over time) is calculated by summing all possible paths for each variable from time 1 to time 2, divided by the total covariance of ADHD and cognitive variable at time 2. Covariance can be transmitted via the stability paths (paths *a* or *b*), via the cross-lagged paths (paths *c* or *d*), and via correlation paths (paths r*f*_1_, r*nf*_1_). As standard procedure, we used unstandardised path coefficients to calculate these pathways or routes (e.g., Burt et al., 2005; Supplemental Table S3).

## Results

### Phenotypic Cross-Lagged and Stability Paths

**Figure 2.**
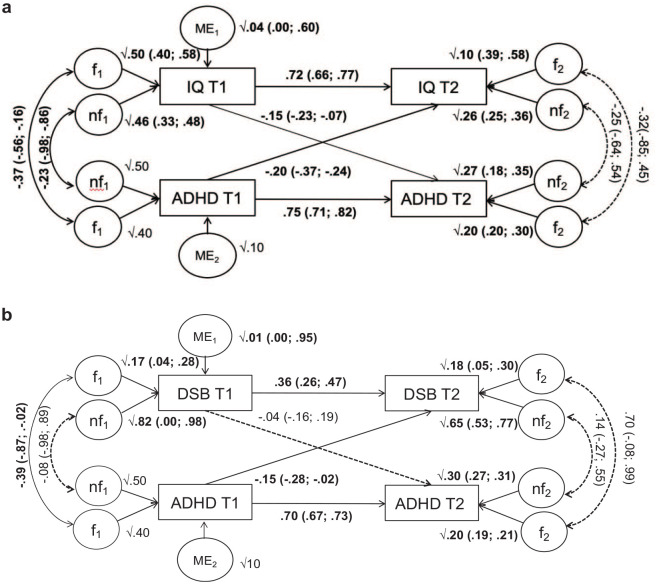
(a and b) Path diagram with standardized effects for ADHD and IQ (a) and for ADHD and Digit Span Backward (DSB; b). *Note*. Dotted lines represent non-significant effects; significant estimates (95% CI excluding zero) are reported in bold. To correct for sample selection, the *f, nf*, and ME effects for ADHD *T*1 are fixed to 40%, 50%, and 10%, respectively, the stability path from ADHD *T*1 to *T*2 is fixed to 0.70, and whereas we allow the residual *f*_2_ and *nf*_2_ effects for ADHD at *T*2 to be estimated, we constrain the total (transmitted and residual) *f* and *nf* to be 40% and 60%, respectively. *f =* familial effects; *nf* = non-familial effects; ME *=* measurement error; *T*1 = time 1; *T*2 = time 2.

ADHD and IQ showed significant reciprocal associations over time as shown by the 95% confidence intervals of the cross-lagged paths (Figure 2a). The cross-lagged path between ADHD at time 1 and DSB at time 2 was significant, while the cross-lagged path between DSB at time 1 and ADHD at time 2 was non-significant (Figure 2b). Cross-lagged paths between ADHD diagnosis at time 1 and all other cognitive variables at time 2, as well as between these associations in the opposite direction (cognitive variables at time 1 predicting ADHD at time 2), were non-significant (Figures S1–S5). Stability paths for all cognitive variables were significant and moderate to large (Figures 2 and Figures S1–S5). The stability path for ADHD was significant in each model and ranged from 0.66 to 0.73 (see Figures S1–S5).

### Time-Specific Familial and Non-Familial Influences and Correlations

Familial and non-familial influences on each variable at both time points are reported in Supplemental Table S4.

At time 1, the familial and non-familial correlations between ADHD diagnosis and IQ, DSB, DSF, MRT in both task conditions, and RTV in both task conditions are reported in Table 1. At time 2, the correlations between variables are estimated as residuals, indicating the association between the variables over and above their relationship at time 1 (Table 1). At time 1, all familial correlations between ADHD and each cognitive variable were significant and showed moderate associations (ranging from −0.39 to −0.32 for IQ, DSF, DSB; and from 0.33 to 0.48 for MRT and RTV in both task conditions; Table 1). The non-familial correlation between ADHD and RTV in the fast-incentive condition and between ADHD and IQ was significant although small (0.25 and −0.23, respectively), while non-familial correlations between ADHD and MRT in both task conditions and RTV in the baseline condition were significant and showed modest to large associations (ranging from 0.30 to 0.50 for MRT in both task conditions and RTV in the baseline condition). Non-familial correlations between ADHD and DSF and DSB at time 1 were non-significant.

**Table 1.** Familial and Non-Familial Correlations Between ADHD Diagnosis and Cognitive Variables at Baseline (Time 1) and At Follow-Up (Time 2).

ADHD at time 1		
	Familial [95% CI]	Non−familial [95% CI]
IQ *T*1	**−0.37 [−0.56, −0.16]**	−**0.23 [−0.98, −0.86]**
DSF *T*1	**−0.32 [−0.61, −0.05]**	−0.17 [−0.89, 0.01]
DSB *T*1	**−0.39 [−0.87, −0.02]**	−0.08 [−0.98, 0.89]
MRT baseline *T*1	**0.40 [0.14, 0.62]**	**0.50 [0.25, 0.80]**
MRT fast-incentive *T*1	**0.33 [0.04, 0.62]**	**0.30 [0.14, 0.99]**
RTV baseline *T*1	**0.48 [0.17, 0.90]**	**0.46 [0.22, 0.93]**
RTV fast-incentive *T*1	**0.43 [0.18, 0.91]**	**0.25 [0.10, 0.99]**
ADHD at time 2		
	Residual familial [95% CI]	Residual non-familial [95% CI]
IQ *T*2	−0.32 [−0.85, 0.45]	−0.25 [−0.64, 0.54]
DSF *T*2	−0.43 [−0.69, 0.26]	−0.18 [−0.57, 0.25]
DSB *T*2	0.70 [−0.08, 0.99]	0.14 [−0.27, 0.55]
MRT baseline *T*2	0.37 [−0.38, 0.97]	0.08 [−0.27, 0.42]
MRT fast-incentive *T*2	−0.02 [−0.79, 0.66]	−0.28 [−0.60, 0.06]
RTV baseline *T*2	0.59 [−0.26, 0.95]	0.13 [−0.23, 0.47]
RTV fast-incentive *T*2	0.68 [−0.09, 0.99]	0.06 [−0.31, 0.44]

*Note*. DSF = digit span forward; DSB = digit span backward; MRT = mean reaction time; RTV = reaction time variability; ME = measurement error; *T*1 = time 1; T2 = time 2.

Bold = *p* < .050.

All residual familial and non-familial correlations were non-significant at time 2 (Table 1). This suggests that the familial and non-familial associations between ADHD and these cognitive measures at time 2 are predominantly due to pre-existing associations at time 1 rather than to new familial and non-familial influences emerging at time 2. Non-significance of the residual familial and non-familial correlations is indicated by confidence intervals spanning zero. It should be noted that this is sometimes the case even if the point estimates are quite large (here, DSB and RTV in both conditions). The fact that such large effects (e.g., 0.70) have still not have been picked up as significant is due to them concerning correlations between residual variances at time 2, which are much smaller than time 1, which in turn reduces statistical power to detect significant results (as illustrated by the wide confidence intervals).

### Familial and Non-Familial Associations Between ADHD and Cognitive Variables Over Time

Familial and non-familial associations between ADHD and cognitive variables over time are reported in Table 2.

**Table 2.** Familial and Non-Familial Associations Between ADHD and Cognitive Variables Specific for Time 2 and Transmitted From Time 1.

	ADHD at time 2	
	Familial	Non-familial
IQ *T*2		
Total covariance	0.41	0.59
Specific effects at *T*2	0.17 (38%)	0.27 (49%)
Contribution from *T*1	**0.28 (62%)**	**0.28 (51%)**
DSF *T*2		
Total covariance	0.61	0.39
Specific effects at *T*2	0.42 (69%)	0.30 (77%)
Contribution from *T*1	**0.19 (31%)**	0.09 (23%)
DSB *T*2		
Total covariance	0.60	0.40
Specific effects at *T*2	0.42 (70%)	0.30 (75%)
Contribution from *T*1	**0.18 (30%)**	0.10 (25%)
MRT baseline *T*2		
Total covariance	0.48	0.52
Specific effects at *T*2	0.23 (48%)	0.12 (23%)
Contribution from *T*1	**0.25 (52%)**	**0.40 (77%)**
MRT fast-incentive *T*2		
Total covariance	0.49	0.51
Specific effects at *T*2	0.25 (51%)	0.17 (33%)
Contribution from *T*1	**0.24 (49%)**	**0.34 (67%)**
RTV baseline *T*2		
Total covariance	0.47	0.52
Specific effects at *T*2	0.27 (57%)	0.16 (31%)
Contribution from *T*1	**0.20 (43%)**	**0.36 (69%)**
RTV fast-incentive *T*2		
Total covariance	0.64	0.36
Specific effects at *T*2	0.47 (73%)	0.09 (25%)
Contribution from *T*1	**0.17 (27%)**	**0.27 (75%)**

*Note*. DSF = digit span forward; DSB = digit span backward; MRT = mean reaction time; RTV = reaction time variability; ME = measurement error; *T*1 = time 1; *T*2 = time 2.

Bold = *p* < .05.

At time 2 a high proportion of stable effects emerged in the familial covariation between ADHD and IQ (62% stable effects). The familial covariation between ADHD and DSB, between ADHD and DSF and between ADHD and RTV in the fast incentive condition showed a high degree of time-specific effects (ranging between 69% and 73% stable effects). An equal proportion of stable versus time-specific effects emerged between ADHD and MRT in the baseline condition (48% time-specific effects vs. 52% stable effects), between ADHD and MRT in the fast-incentive condition (51% time-specific effects vs. 49% stable effects), and between ADHD and RTV in the baseline condition (57% time-specific effects vs. 43% stable effects).

Non-familial influences between ADHD and MRT in both conditions, and between ADHD and RTV in both conditions, showed a high proportion of stable effects at time 2 (ranging between 67% and 77% stable effects). Time-specific non-familial influences emerged in the covariation between ADHD and DSB, and ADHD and DSF ( 75% and 77% time-specific effects respectively). An equal proportion stable versus time-specific effects emerged in the non-familial covariation between ADHD and IQ (49% time-specific effects vs. 51% stable effects).

Although the covariation between ADHD and cognitive variables at time 2 showed both stable and time-specific effects, the residual estimated correlations between ADHD and each cognitive variable were non-significant, suggesting that only stable effects significantly influence the association between ADHD and the cognitive measures at time 2.

## Discussion

Using longitudinal assessments of ADHD diagnosis and cognitive performance in affected and control sibling pairs, we found evidence of reciprocal associations between ADHD diagnosis and IQ over time. Further, ADHD diagnosis in childhood and adolescence predicted impaired working memory (DSB), but not short-term memory (DSF), response variability (RTV), or response speed (MRT), at follow-up 6 years later. The shared familial and non-familial effects influencing the associations between ADHD and cognitive measures in childhood showed stability over time, although significant time-specific familial and non-familial influences emerged for ADHD and each cognitive impairment at follow-up.

Our finding that ADHD diagnosis in childhood predicts impaired working memory in adolescence and young adulthood, over and above their relationship in childhood, suggests that childhood ADHD has a negative impact on future working memory performance. One possible interpretation is that children with ADHD, compared to children without the disorder, have more difficulties paying attention while performing daily and academic tasks, which may make encoding task information and retrieving it from working memory more difficult (Lenartowicz et al., 2019). As the complexity of daily and academic tasks increases during development, the inattentive symptoms associated with ADHD might contribute to the worsening of working memory over time, explaining the longitudinal association found in this study.

We further show evidence of reciprocal association between ADHD and IQ over time: ADHD in childhood predicted lower IQ scores at follow-up, and vice versa. This result extends our previous uni-directional analysis that showed lower IQ at baseline predicting later ADHD symptoms and impairment using a subset of the present sample (only the childhood ADHD group; Cheung et al., 2015). The evidence of reciprocal association between ADHD and IQ over time was also reported in our separate population-based study, which showed that ADHD symptoms and verbal and performance IQ reciprocally predicted each other over time (Rommel et al., 2015). Taken together, these results converge in showing that ADHD and IQ mutually influence each other during development and suggest the possibility that IQ moderates ADHD outcomes (Cheung et al., 2015). For instance, individuals with higher IQ may develop better coping strategies to deal with their ADHD symptoms, compared to those with lower IQ. At the same time, ADHD symptoms may negatively interfere with learning at school, reducing the opportunities for cognitively benefiting from education (Ritchie & Tucker-Drob, 2018). Future studies would benefit from also investigating general cognitive ability using “culture-free” IQ tests, in order to detect underlying cognitive potential not affected by culture and learning.

We further provide evidence that ADHD was not a predictor of DSF, MRT, and RTV. This pattern suggests that impairments in DSF, MRT, and RTV co-occur with ADHD without influencing its outcome over time. Of note, we previously showed that RTV at follow-up was comparable between those with remitted ADHD and participants without ADHD at baseline and follow-up, but impaired in individuals whose ADHD persisted. This evidence suggests that RTV might be a marker of ADHD remission, while this pattern was not observed for DSF and DSB as those measures were not sensitive to ADHD persistence/remission (Cheung et al., 2016). Despite all measures being cross-sectionally associated with ADHD, the finding that RTV is a marker of ADHD remission, together with the current finding that ADHD and RTV co-occur with no reciprocal influence over time, highlights that RTV may represent an objective measure of the attention fluctuations related to the core ADHD symptoms.

We further assessed the stability or change of the familial and non-familial influences on the association between ADHD and cognitive measures over time. Our results show that the associations between ADHD and each investigated cognitive variable at follow-up was attributable to stable familial and non-familial influences from baseline (except for the non-familial influences for DSB and DSF as the association with ADHD for these variables at baseline was non-significant). This result was supported by the significance of the correlations between ADHD and each cognitive variable at baseline and at follow-up in the cross-lagged model, since only significant correlations at baseline can show stability over time, while significance of the correlations at follow-up would suggest significance of new time-specific effects at follow-up. Given that the associations between ADHD and each investigated cognitive variable at follow-up was non-significant, our result suggests that the new etiological influences emerging after 6 years do not contribute significantly to the association between ADHD and cognitive performance and that these associations are accountable by stable effects only. However, given the wide confidence intervals, these familial and non-familial correlations at follow-up should be interpreted cautiously, and future studies using larger samples are required to further examine these associations between ADHD and cognitive functioning over time.

The following limitations should be considered when interpreting these findings. Given that this study focuses on sibling data only, it allowed the investigation of familial and non-familial effects, but we could not directly estimate the contribution of genetic factors. However, as previous evidence suggests a limited role for shared environmental influences on either ADHD (Burt, 2009; Burt et al., 2012) or cognitive markers (Anokhin et al., 2008; Kuntsi et al., 2013), the familial overlap between ADHD and such markers is expected to largely reflect genetic influences. Another limitation of this study is the wide age range. Future studies using more restricted age ranges and, ideally, multiple follow-ups should replicate and extend the current results. Finally, despite the cross-lagged model being a valid tool for assessing the directional influences variables have on each other over time, it does have certain limitations. First, its underlying assumptions of synchronicity and stationarity imply that the variables at each time point were assessed simultaneously, and that the etiological influences would stay the same across different time points (Kenny, 1975). Although the diagnosis of ADHD was assessed during a wide developmental window (between ages 6 and 17 years), the genetic version of the cross-lagged model used in our analyses allows us to assess new familial and non-shared environmental influences at follow-up. Therefore, violation of these assumptions is unlikely in this study. Another common limitation of the cross-lagged design is the assumption that there are no errors which can bias the stability and/or cross-lagged path estimates. To account for this bias, we specified measurement errors for each variable in our model based on previous studies. Lastly, the model assumes that there are no differences in the measured traits between and within people over time. These differences are often captured by specifying intercepts and slopes in cross-lagged models (Hamaker et al., 2015). However, more than two time points are typically expected for such cross-lagged genetic models to be identified, hence these could not be specified in the present analyses. Future analyses would be improved by using data collected at more than two time points.

In conclusion, our findings indicate reciprocal association between ADHD and IQ over time and that childhood ADHD predicts future working memory deficits in adolescence and young adulthood, but not future deficits in attention fluctuation, response speed, and short-term memory which instead co-occur with ADHD without influencing its outcome over time. We further provide evidence of stability of familial and non-familial effects influencing the association between ADHD and cognitive measures over time, which requires replications in bigger samples. Based on these findings, future studies may investigate how early interventions can best reduce the negative interference with learning at school and increase the opportunities for individuals with ADHD to cognitively benefit more fully from education.

## Supplemental Material

sj-docx-1-jad-10.1177_10870547231159908 – Supplemental material for The Etiological and Predictive Association Between ADHD and Cognitive Performance From Childhood to Young Adulthood Click here for additional data file. Supplemental material, sj-docx-1-jad-10.1177_10870547231159908 for The Etiological and Predictive Association Between ADHD and Cognitive Performance From Childhood to Young Adulthood by Isabella Vainieri, Giorgia Michelini, Celeste H. M. Cheung, Olakunle A. Oginni, Philip Asherson, Frühling Rijsdijk and Jonna Kuntsi in Journal of Attention Disorders
