# Supplementary material for: The Etiological and Predictive Association Between ADHD and Cognitive Performance From Childhood to Young Adulthood
Source: J Atten Disord. 2023 Apr 11;27(7):709–20. doi: 10.1177/10870547231159908 (PMC10173328; doi:10.1177/10870547231159908)
Supplement: sj-docx-1-jad-10.1177_10870547231159908 – Supplemental material for The Etiological and Predictive Association Between ADHD and Cognitive Performance From Childhood to Young Adulthood [file sj-docx-1-jad-10.1177_10870547231159908.docx]

**The aetiological and predictive association between ADHD and cognitive impairments from childhood to young adulthood**

**Supplementary information**

**Further information on participants**

Participants were recruited among those who had taken part in our previous research (UK-London sub-sample of the International Multicentre ADHD Genetics (IMAGE) project (Chen et al.,2008; Kuntsi, Neale et al., 2006b; Kuntsi et al., 2010;) were invited to take part in this study.

At follow-up (on average 6 years after initial assessment), participants were contacted by telephone and scheduled for a single testing session including clinical, cognitive and EEG assessments. Retention rate at follow-up was 77%. The sample retained at follow-up consisted of 404 participants, including 226 participants from ADHD sibling pairs (each including one ADHD proband and one affected or unaffected sibling), and 178 participants from control sibling pairs (both without ADHD) who had taken part in our previous research (Chen et al., 2008; Kuntsi et al., 2010). From these 404 participants, six siblings of ADHD probands were excluded as their diagnostic status could not be determined due to missing parent-reported data on impairment. Seven childhood ADHD probands were excluded for not having combined-type ADHD in childhood or due to equipment failure. The sample, after the exclusion of participants with missing data due to equipment failure or missing information on ADHD rating, consisted of 391 participants. 99 participants with ADHD and 100 unaffected siblings (69 full sibling pairs, 61 singletons), 23 remitters (5 full sibling pairs , 13 singletons), and 169 control siblings (76 full sibling pairs, 17 singletons).

Among those with childhood ADHD at baseline, 87 continued to meet clinical (DSM- IV) levels of ADHD symptoms and impairments (ADHD persisters) and 23 were below the clinical cut-off at follow-up (ADHD remitters). Three siblings of ADHD probands were unaffected in childhood but met DSM-IV ADHD criteria at follow-up. Nine controls met ADHD criteria at follow-up based on parent-reported ADHD rating.

**Supplementary Tables**

**Table S1.** Sample demographics divided by group, with test for group difference

|  | ADHD probands  (n= 99) | Unaffected siblings  (n=100) | Remitters  (n=23) | Controls  (n=169) | p |
| --- | --- | --- | --- | --- | --- |
| SEX (M:F) | 84:15 | 53:47 | 23:0 | 129:40 | **0.02*** |
| Age | 18.34 (3.03) | 18.56 (3.33) | 18.89 (3.06) | 17.75 (2.17) | 0.07 |

Note: group differences on gender were tested via Chi-square test; group differences on age were tested with regression models. Bold = p<0.050.

**Table S2.** Descriptive statistics for all study variables at both time points

|  | ADHD | | non-ADHD | |
| --- | --- | --- | --- | --- |
|  | T1  Mean (SD) | T2  Mean (SD) | T1  Mean (SD) | T2  Mean (SD) |
| IQ | 99.88 (14.84) | 96.56 (15.54) | 106.29 (13.81) | 104.04 (13.52) |
| DSF | 8.29 (2.13) | 9.27 (2.08) | 9.13 (2.15) | 10.18 (2.13) |
| DSB | 4.90 (1.99) | 6.29 (2.43) | 5.51 (2.01) | 7.48 (2.53) |
| MRT Baseline | 822.18 (267.66) | 637.99 (177.85) | 686.52 (243.34) | 553.71 (130.79) |
| MRT Fast-incentive | 587.21 (162.03) | 483.36 (105.66) | 524.06 (159.84) | 427.17 (63.98) |
| RTV Baseline | 403.35 (330.18) | 208.88 (245.75) | 220.02 (217.95) | 116.37 (108.02) |
| RTV Fast-incentive | 183.85 (123.29) | 100.11 (94.05) | 131.88 (119.46) | 63.93 (46.82) |

Note: Digit Span Forward, DSF; Digit Span Backward, DSB; Mean reaction Time, MRT; Reaction Time Variability, RTV; Time 1, T1; Time 2, T2.

**Table S3.**  Unstandardized coefficients of the cross-lagged model of ADHD with IQ and ADHD and DBS

|  | IQ | DSB |
| --- | --- | --- |
| f_11_ | 1.041 | 0.748 |
| f_12_ | 0.40 (fixed) | 0.40 (fixed) |
| nf_11_ | 0.661 | 1.665 |
| nf_12_ | 0.60 (fixed) | 0.60 (fixed) |
| rf_1_ | -0.338 | -0.392 |
| rnf_1_ | -0.340 | -0.080 |
| a | 0.722 | 0.481 |
| b | 0.060 | 0.412 |
| c | -0.169 | -0.383 |
| d | -0.723 | -0.022 |
| ME | 0.707 | -0.003 |
| Total variance T2 | 1.89 | 6.41 |

Note: Digit Span Backward, DSB; Measurement error, ME. Familial and non-familial influences for ADHD at T1 fixed to population-based values.

**Time-specific familial and non-familial influences**

At time 1, familial influences were small for DSB, MRT in the fast incentive condition, and RTV in both conditions (ranging from 0.17 to 0.28), and moderate to large for IQ, DSF, and MRT in the baseline condition (ranging from 0.29 to 0.50). Non-familial influences were moderate to large at time 1 for all variables (ranging from 0.44 to 0.82). As reported in the methods section, familial and non-familial influences for ADHD at time 1 and for ADHD at time 2 were fixed to population-based parameters given the selected nature of this sample (40% and 60%, respectively). The total familial variance at time 2 was small for DSB, MRT and RTV in both conditions (ranging from 0.18 to 0.25), while the total familial variance at time 2 for IQ and DSF was moderate (0.41 and 0.31, respectively). The total non-familial variance at time 2 was moderate to large for all variables (ranging from 0.55 to 0.82). Residual familial and non-familial influences at time 2 indicate the familial and non-familial contributions that are independent of the familial and non-familial influences transmitted from time 1 (time-specific for time 2). Time-specific familial contributions for each variable at time 2 were small and ranged from 0.12 to 0.22, while time-specific non-familial contributions at time 2 were moderate to large for all variables (ranging from 0.48 to 0.70) and small for IQ (0.26) (residuals, Table 1). Overall, new familial and non-familial influences on cognitive performance and ADHD emerged at time 2, which were not explained by familial and non-familial influences at time 1. The proportion of the total familial and non-familial variance at time 2 explained by time-specific influences was high for all variables (ranging from 64% to 92% for familial influences, and from 70% to 91% for non-familial influences). Only for IQ, a small proportion of the total familial variance was explained by time-specific influences (24%), while the total non-familial variance explained by time-specific influences was moderate (43%). Further details are reported in table S4.

**Table S4.** Familial and non-familial influences at both time points and proportion of total variance due to residual familial and non-familial influences

|  | T1 | |  | T2 | |
| --- | --- | --- | --- | --- | --- |
|  | ***Familial***  ***95% CI*** | ***Non-familial***  ***95% CI*** |  | ***Familial***  ***95% CI*** | ***Non-familial***  ***95% CI*** |
| IQ | **0.50 (0.40; 0.60)** | **0.46 (0.33; 0.48)**  **ME=0.04** | *Total variance T2* | **0.41 (0.37; 0.52)** | **0.59 (0.47; 0.62)** |
|  |  |  | *Residual* | **0.10 (0.39; 0.58)**  **(24%)** | **0.26 (0.25; 0.36)**  **(44%)** |
| DSF | **0.29 (0.17; 0.39)** | **0.49 (0.00, 0.82)**  **ME= 0.22** | *Total variance T2* | **0.31 (0.18; 0.41)** | **0.69 (0.58; 0.81)** |
|  |  |  | *Residual* | **0.22 (0.10, 0.32)**  **(71%)** | **0.48 (0.38; 0.58)**  **(70%)** |
| DSB | **0.17 (0.04; 0.28)** | **0.82 (0.00; 0.98)**  **ME = 0.01** | *Total variance T2* | **0.22 (0.09; 0.34)** | **0.78 (0.65; 0.90)** |
|  |  |  | *Residual* | **0.18 (0.05; 0.30)**  **(82%)** | **0.65 (0.53; 0.77)**  **(83%)** |
| MRT Baseline | **0.33 (0.20; 0.44)** | **0.44 (0.04, 0.79)**  **ME = 0.23** | *Total variance T2* | **0.25 (0.14; 0.35)** | **0.75 (0.64; 0.85)** |
|  |  |  | *Residual* | **0.16 (0.05; 0.27)**  **(64%)** | **0.55 (0.44; 0.67)**  **(75%)** |
| MRT Fast-incentive | **0.28 (0.14; 0.40)** | **0.71 (0.07; 0.85)**  **ME = 0.01** | *Total variance T2* | **0.25 (0.12; 0.35)** | **0.75 (0.64; 0.87)** |
|  |  |  | *Residual* | **0.18 (0.06; 0.29)**  **(75%)** | **0.60 (0.48; 0.72)**  **(80%)** |
| RTV Baseline | **0.21 (0.06; 0.33)** | **0.55 (0.04, 0.92)**  **ME = 0.24** | *Total variance T2* | **0.18 (0.13; 0.30)** | **0.82 (0.69; 0.93)** |
|  |  |  | *Residual* | **0.14 (0.01, 0.27)**  **(83%)** | **0.70 (0.58; 0.83)**  **(87%)** |
| RTV Fast-incentive | **0.17 (0.10; 0.31)** | **0.80 (0.68; 0.99)**  **ME = 0.03** | *Total variance T2* | **0.24 (0.09; 0.37)** | **0.76 (0.62; 0.98)** |
|  |  |  | *Residual* | **0.22 (0.06; 0.36)**  **(92%)** | **0.69 (0.56; 0.84)**  **(91%)** |
| ADHD | 0.40 (fixed) | 0.50 (fixed)  ME=0.10 (fixed) | *Total variance T2* | 0.40 (fixed) | 0.60 (fixed) |
|  |  |  | *Residual* | **0.20 (0.19; 0.30)** | **0.30 (0.18; 0.32)** |

Note: Digit Span Forward, DSF; Digit Span Backward, DSB; Mean Reaction Time, MRT; Reaction Time Variability, RTV; Attention-deficit/hyperactivity disorder, ADHD; Measurement Error, ME; Time 1, T1; Time 2, T2. Familial and non-familial influences for ADHD at T1 fixed at 0.40 (and total effects for ADHD at T2 constrained to 0.60) population values. Familial and non-familial influences for ADHD at T2 free (here reported as the mean of the influences across models). *Residual* indicates the familial and non-familial contributions independent of the familial and non-familial contributions transmitted from T1 (time specific for time 2). The proportion of total variance due to residual is given in brackets. Bold = p<0.05, 95% CI not including zero.

**Supplementary Figures**

**Figure S1.** Path diagram with standardized effects for ADHD status and Digit Span Forward (DSF). Note: familial effects, f; non-familial effects, nf. Measurement error, ME; Attention-deficit/hyperactivity disorder, ADHD; Time 1, T1; Time 2, T2. Dotted lines represent non-significant estimates; significant estimates (95% CI excluding zero) are reported in bold.


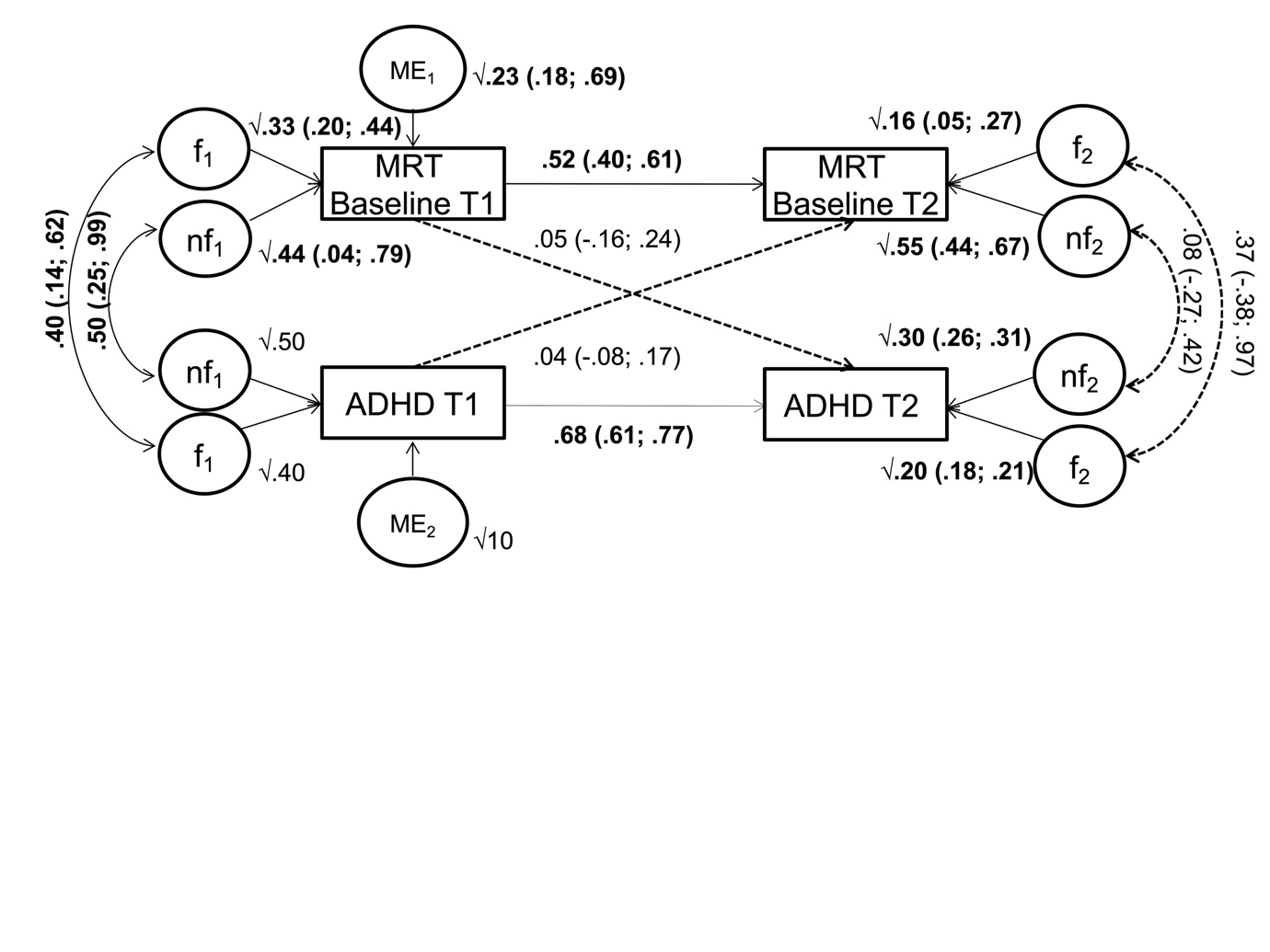


**Figure S2.** Path diagram with standardized effects for ADHD status and Mean Reaction Time (MRT) in the Baseline condition of the Fast-task. Note: familial effects, f; non-familial effects, nf. Measurement error, ME; Attention-deficit/hyperactivity disorder, ADHD; Time 1, T1; Time 2, T2. Dotted lines represent non-significant results and thick lines represent significant estimates; significant estimates (95% CI excluding zero) are reported in bold.


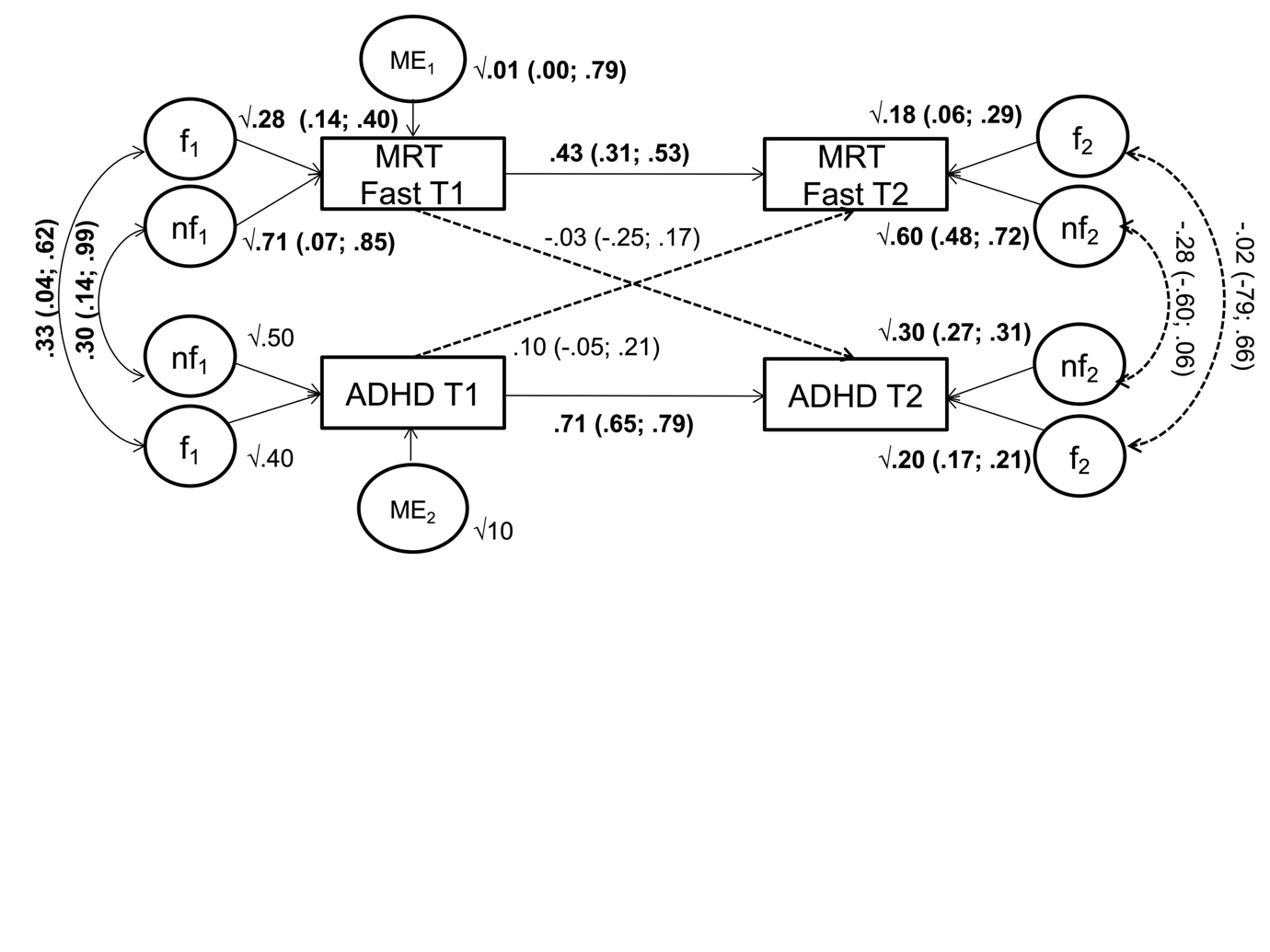


**Figure S3.** Path diagram with standardized effects for ADHD status and Mean Reaction Time (MRT) in the Fast-incentive condition of the Fast-task. Note: familial effects, f; non-familial effects, nf. Measurement error, ME; Attention-deficit/hyperactivity disorder, ADHD; Time 1, T1; Time 2, T2. Dotted lines represent non-significant results and thick lines represent significant estimates; significant estimates (95% CI excluding zero) are reported in bold.


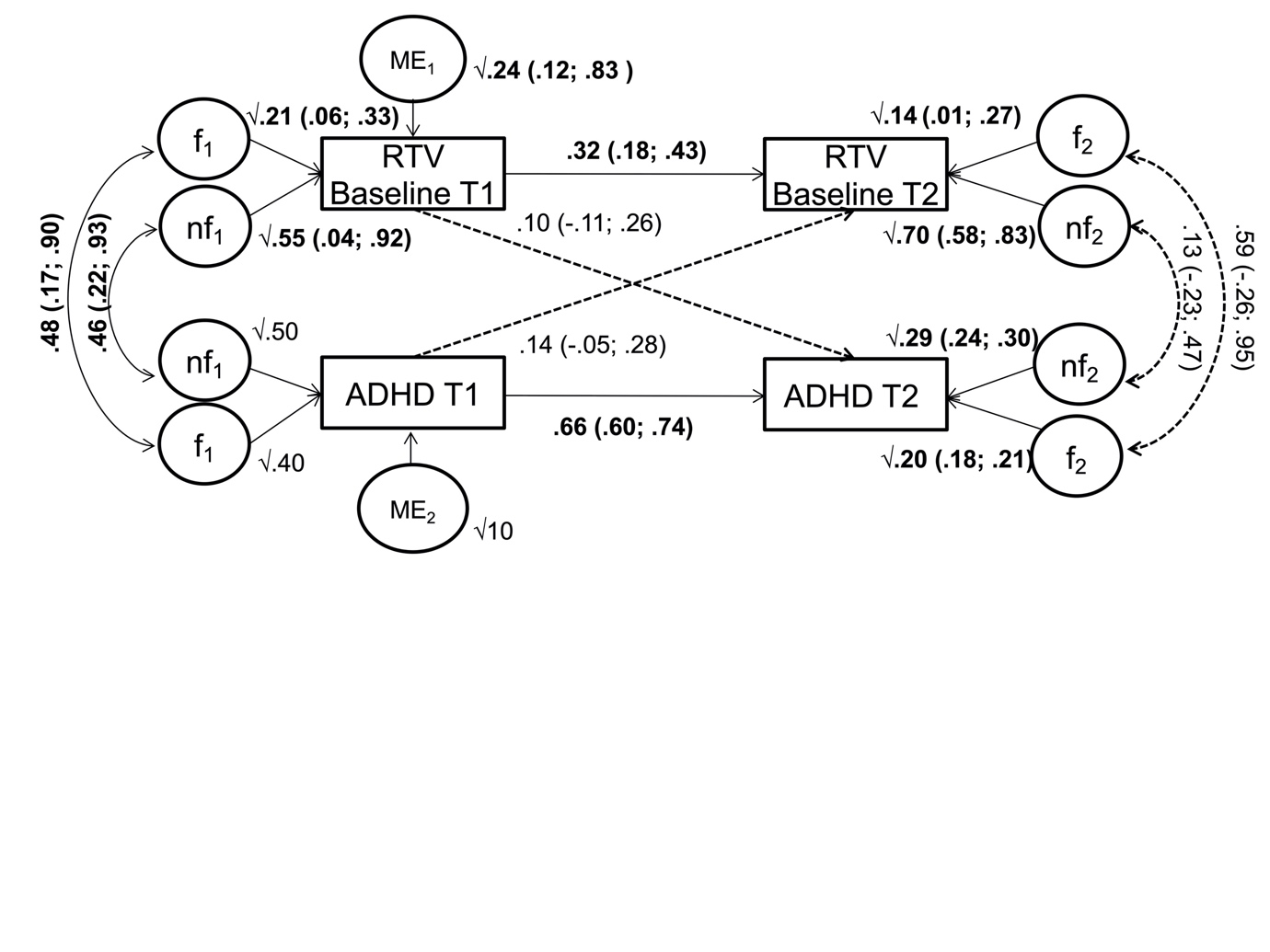


**Figure S4.** Path diagram with standardized effects for ADHD status and Reaction Time Variability (RTV) in the Baseline condition of the Fast-task. Note: familial effects, f; non-familial effects, nf. Measurement error, ME; Attention-deficit/hyperactivity disorder, ADHD; Time 1, T1; Time 2, T2. Dotted lines represent non-significant results and thick lines represent significant estimates; significant estimates (95% CI excluding zero) are reported in bold.


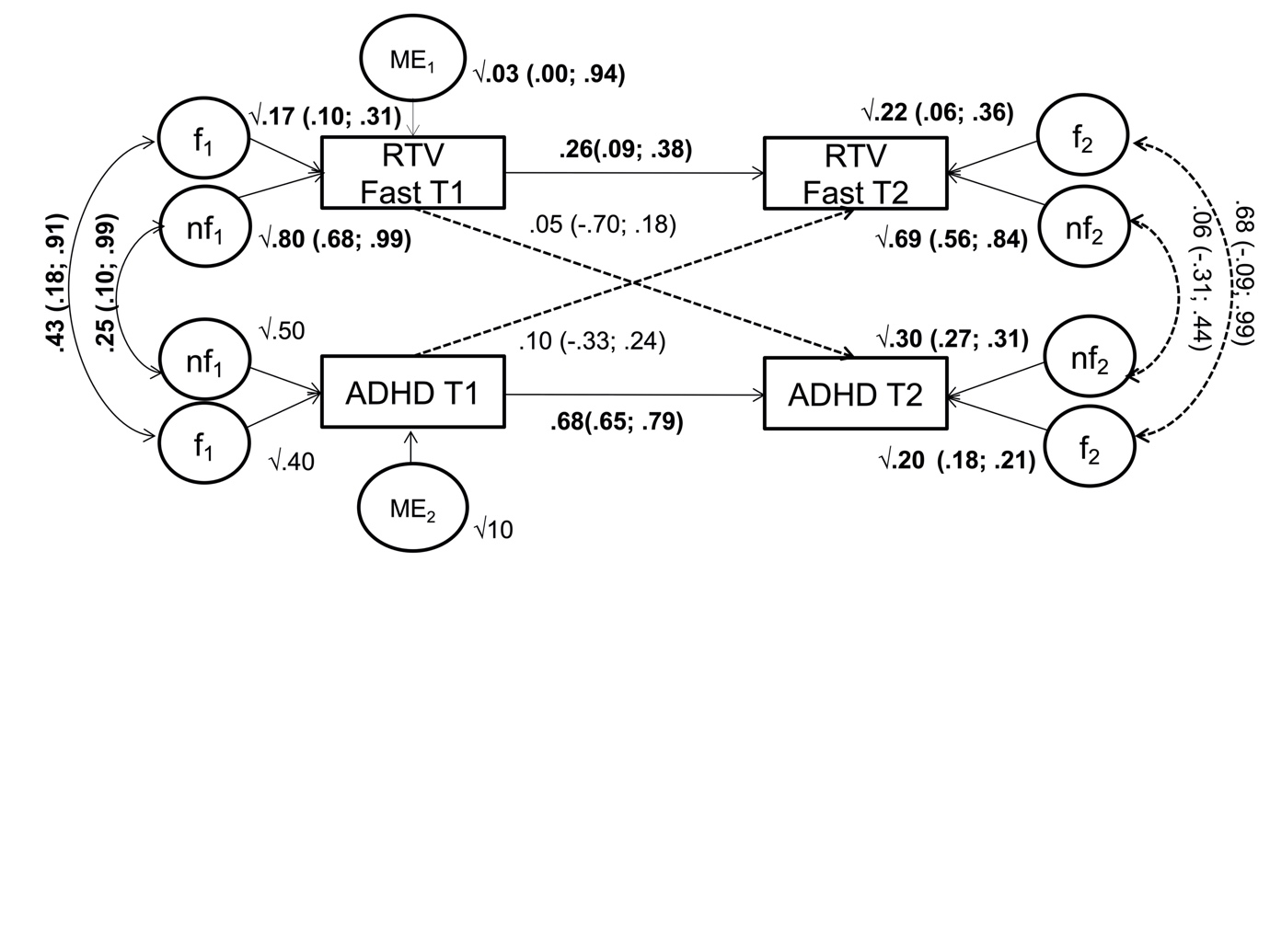


**Figure S5.** Path diagram with standardized effects for ADHD status and Reaction Time Variability (RTV) in the Fast-incentive condition of the Fast-task. Note: familial effects, f; non-familial effects, nf. Measurement error, ME; Attention-deficit/hyperactivity disorder, ADHD; Time 1, T1; Time 2, T2. Dotted lines represent non-significant results and thick lines represent significant results; estimates; significant estimates (95% CI excluding zero) are reported in bold.
